# Sleep Disturbances and Pain Subtypes in Parkinson’s Disease

**DOI:** 10.3390/medicina61040591

**Published:** 2025-03-26

**Authors:** Stefania Diaconu, Bianca Ciopleias, Anca Zarnoveanu, Cristian Falup-Pecurariu

**Affiliations:** 1 Department of Neurology, County Clinic Hospital, 500326 Brasov, Romania; stefi_diaconu@yahoo.com (S.D.); crisfp100@yahoo.co.uk (C.F.-P.); 2 Faculty of Medicine, Transilvania University, 500019 Brasov, Romania; opritoiubianca@yahoo.com

**Keywords:** sleep disturbances, pain subtypes, Parkinson’s disease, non-motor symptoms, sleep quality

## Abstract

*Background and Objectives*: Sleep and pain are non-motor symptoms encountered frequently in Parkinson’s disease (PD). Several subtypes of pain have been identified in PD, with different associations with other non-motor symptoms. To evaluate the prevalence of various subtypes of pain in a PD cohort and their associations with sleep disturbances and quality of sleep. *Materials and Methods*: In this study, 131 consecutive PD patients were assessed, focusing on pain and sleep using several validated scales and questionnaires. *Results*: According to KPPQ, the most reported types of pain were musculoskeletal pain (82.44%), nocturnal pain (58.77%), and radicular pain (55.72%). “Bad sleepers” (PSQI score > 5) reported significantly more pain than “good sleepers” regarding all KPPS subdomains, with statistically significant differences observed in the following domains: musculoskeletal pain (5.48 ± 3.50 vs. 2.70 ± 2.67, *p* < 0.001), chronic pain, specifically central pain (1.19 ± 2.01 vs. 0.15 ± 0.71, *p* = 0.004), nocturnal pain, specifically pain related to akinesia (2.26 ± 2.74 vs. 0.64 ± 1.22, *p* = 0.001), and radicular pain (4.35 ± 4.20 vs. 2.45 ± 3.55, *p* = 0.022). The prevalence of sleep disturbances was higher in patients with nocturnal pain (odds = 1.165, 95% CI: 1.064–1.276, *p* = 0.001), orofacial pain (odds = 1.108, 95% CI: 1.051–1.167, *p* < 0.001), and radicular pain (odds = 1.015, 95% CI: 1.015–1.149, *p* = 0.015). *Conclusions*: Pain is common in PD patients with sleep disorders. Identifying specific types of pain that are associated with sleep disorders and their correct management may improve sleep quality.

## 1. Introduction

Sleep disorders are frequently encountered in Parkinson’s disease (PD), with a broad spectrum of manifestations that impact the quality of life of these patients [1,2,3]. Several factors are involved in the occurrence of sleep disturbances in PD, such as poor sleep hygiene, the neurodegenerative processes that impair the physiological sleep regulatory mechanisms, side effects of medication, and the association with other motor and non-motor symptoms [4,5]. Pain is another bothersome symptom in PD, with a high prevalence [6] and negative impact on quality of sleep [7,8].

Pain can be subdivided into several types in patients with PD, namely, musculoskeletal pain, chronic pain associated with PD (central and visceral), pain related to motor fluctuations responsive to dopaminergic therapy (dyskinesia-related pain, pain associated with dystonia during “off” periods, generalized pain during “off” periods), nocturnal pain (associated with restless legs syndrome or difficulty turning in bed), “coat-hanger” pain (in the shoulder area), orofacial pain (temporomandibular joint pain, associated with bruxism, glossodynia, or “burning mouth” syndrome), abdominal pain, and lower limb pain induced by dopaminergic treatment [9]. Central pain is very characteristic in PD, suggesting neuropathic pain that cannot be explained by other causes (musculoskeletal complaints, dystonia, etc.) [7].

Although the interconnection between pain and sleep dysfunctions has been demonstrated in previous research [10], there are few data on specific subtypes of pain and sleep disturbances in PD [11,12]. Therefore, the aims of this study were to identify the prevalence of different subtypes of pain in PD patients and explore the correlations between these subtypes of pain and sleep disturbances and quality of sleep.

## 2. Materials and Methods

### 2.1. Patients and Study Design

This research was designed as a cross-sectional study, in which 131 PD patients were included. The inclusion criteria were as follows: patients with a diagnosis of PD (based on the latest criteria proposed by the Movement Disorders Society (MDS) [13]), absence of severe cognitive dysfunction (MMSE > 10), and willingness to voluntarily participate in this study. We excluded patients with secondary or atypical forms of parkinsonism, patients with psychiatric disturbances, and those with pain of known causes or a history of neurosurgical interventions.

### 2.2. Clinical Assessments

All patients completed a comprehensive assessment that was designed for the assessment of various types of pain in relation to sleep disturbances. Several data were collected: socio-demographic information, age at PD onset, duration of the disease, levodopa equivalent daily dose (LEDD), comorbidities, and treatment regimen, together with specific scales and questionnaires that will be described in the next section. Each patient was assessed in the “ON” state. All participants have given written consent to be included in this study, which was conducted in accordance with the Declaration of Helsinki and approved by the Ethics Committee of University Transilvania of Braşov (1.11/01/2019).

### 2.3. Tools for Assement

Several validated scales and questionnaires were applied for the assessment of motor and non-motor symptoms, particularly for sleep and pain evaluation. The motor status was assessed with the MDS Unified Parkinson’s disease Rating Scale part III (MDS-UPDRS III) [14], Scales for Outcomes in Parkinson’s Disease (SCOPA) [15]—the motor part, Clinical Impression of Severity Index for PD (CISI-PD) [16], and the Hoehn–Yahr (HY) scale. The Mini Mental Examination Test was used to evaluate the global cognitive function. The King’s Parkinson’s Disease Pain Questionnaire (KPPQ) is a pain screening questionnaire specifically designed and validated for PD patients. It consists of 14 items addressing various types of pain, to which the patient can respond with either “yes” or “no”, depending on whether or not they experience that particular type of pain [17]. The KPPS (King’s Parkinson’s Disease Pain Scale) was developed in 2015 by Chaudhuri et al. to describe and characterize pain in detail in PD patients. The total score can range from 0 to 144, with higher values indicating more severe pain [18]. The Pittsburgh Sleep Quality Index (PSQI) [19] is a widely used self-assessment tool for evaluating sleep quality, focusing on the past month. For each question, the patient can choose a response ranging from 0 (no disturbances) to 3 (severe disturbances), with a maximum score of 21 points. A score above 5 is considered indicative of sleep problems [19]. Sleep quality can be quantified based on the total PSQI score as follows: good (≤5), moderate (6–10), and poor (>10). This classification is also used in other studies [20]. The Parkinson’s Disease Sleep Scale-2 (PDSS-2) consists of 15 questions, and patients’ responses are scored from 0 (never) to 4 (very frequently), with a maximum score of 60 points indicating severe sleep disturbances. The scale is easy to administer, relies on self-assessment, and can be used both for screening sleep disorders and quantifying their severity [21]. The Hospital Anxiety and Depression Scale (HADS) contains two subscales (one for anxiety and one for depression) and was used as a self-assessment tool for these symptoms. A score ≥ 11 for each subscale is suggestive of clinically significant symptoms, while a score of 8–10 suggests a mild severity [22]. Restless legs syndrome (RLS) was identified in patients fulfilling all five diagnostic criteria proposed by the International RLS Study Group (IRLSSG) [23].

### 2.4. Data Analysis

Statistical analyses were performed using IBM SPSS Statistics for Windows, Version 26.0. Armonk, NY, USA: IBM Corp. Data were expressed as means ± standard deviation (SD). Probability values of *p* < 0.05 were considered significant. The sample distribution was determined using the Shapiro–Wilk test. Fisher’s exact test and the Mann–Whitney U Test were used to compare the characteristics of pain between groups (“good sleepers” vs. “bad sleepers”). A simple logistic regression model and multiple regression models were created to determine the predictors of sleep disturbances.

## 3. Results

In this study, 131 consecutive PD patients were assessed. The mean age of the patients was 73.79 years; 67 of these patients were females. Demographic and basic clinical data are presented in Table 1. The lowest MMSE score recorded for the patients included in the study was 16.

**Figure 1:**
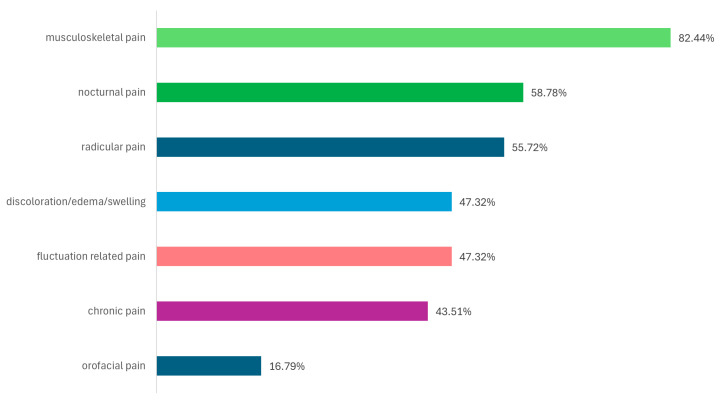
The prevalence of pain subtypes in PD patients (according to the corresponding domains of King’s Parkinson’s Disease Pain Questionnaire).

The prevalence of various subtypes of pain in PD patients is presented in Figure 1. Musculoskeletal pain was most frequent encountered (82.44%), followed by nocturnal (58.77%) and radicular pain (55.72%).

**Figure 2:**
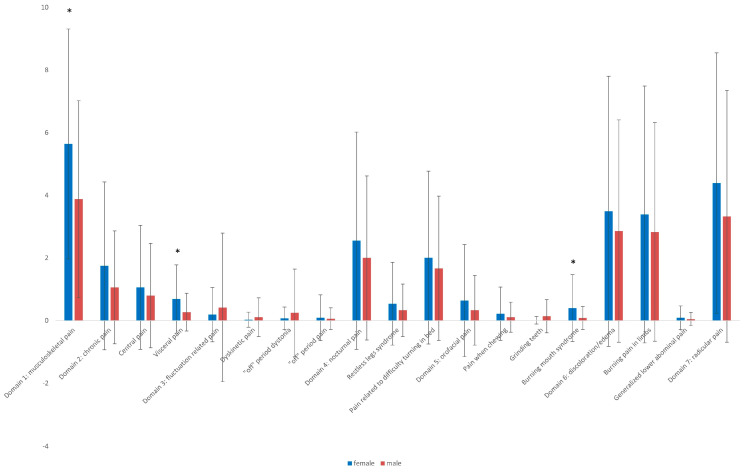
Distribution of mean scores of KPPS (King’s Parkinson’s Disease Pain Scale) domains and subdomains in females vs. males. Significant differences are marked with an asterisk (*).

Figure 2 represents the gender differences between different subtypes of pain, according to KPPS mean scores. Significantly higher mean scores were noted in females in comparison with males: musculoskeletal pain (5.64 ± 3.67 in females, vs. 3.88 ± 3.14 in males, *p* = 0.004), visceral pain (0.69 ± 1.09 in females vs. 0.27 ± 0.60 in males, *p* = 0.007), and burning mouth syndrome (0.40 ± 1.07 in females vs. 0.08 ± 0.37 in males).

The KPPS score is also useful for classifying the severity levels of pain: no pain (KPPS: 0), mild pain (KPPS: 1–17), moderate pain (KPPS: 18–68), and severe pain (KPPS > 68) [18]. Based on these scores, 5 PD patients without pain, 74 with mild pain, and 52 with moderate pain were identified. None of the enrolled PD patients experienced severe pain. Patients with moderate pain had higher mean scores on the SCOPA motor scale (26.25 ± 9.39) compared to those with mild pain (19.40 ± 14.19) or without pain (19.50 ± 7.80); *p* < 0.001. The same pattern was observed for the mean total scores of CISI: 9.62 ± 3.35 for patients with moderate pain, 7.08 ± 3.37 for those with mild pain, and 6.00 ± 3.74 for those without pain; *p* < 0.001. As pain severity increased, higher total scores were observed on the PSQI mean total scores (7.40 ± 2.30 for patients without pain, 8.38 ± 4.94 for those with mild pain, and 11.06 ± 4.81 for those with moderate pain, *p* = 0.007) and PDSS-2 mean total scores (19.20 ± 9.76 for PD patients without pain, 19.26 ± 9.75 for those with mild pain, and 30.38 ± 9.41 for those with moderate pain, *p* < 0.001).

The pain domains (according to the KPPS scale) were analyzed based on the presence or lack of sleep disturbances. Using a PSQI score > 5, PD patients were considered “bad sleepers”, while those with a score ≤ 5 were considered “good sleepers”. The results of the KPPS components based on the presence of sleep disturbances are presented in Table 2. “Bad sleepers” experienced significantly more pain than “good sleepers” across all the KPPS subdomains, with statistically significant differences observed in the following domains: musculoskeletal pain (5.48 ± 3.50 vs. 2.70 ± 2.67, *p* < 0.001), chronic pain, specifically central pain (1.19 ± 2.01 vs. 0.15 ± 0.71, *p* = 0.004), nocturnal pain, specifically pain related to akinesia (2.26 ± 2.74 vs. 0.64 ± 1.22, *p* = 0.001), and radicular pain (4.35 ± 4.20 vs. 2.45 ± 3.55, *p* = 0.022).

In the group of PD patients with pain, a simple logistic regression model (Table 3) highlighted that the odds of sleep disturbances (PDSS-2 score ≥ 18) was higher among patients with nocturnal pain (odds = 1.161, 95% CI: 1.062–1.268, *p* = 0.001), orofacial pain (odds = 1.069, 95% CI: 1.009–1.133, *p* = 0.023), discoloration/edema/swelling (odds = 1.059, 95% CI: 1.004–1.116, *p* = 0.034), and radicular pain (odds = 1.088, 95% CI: 1.024–1.156, *p* = 0.007).

In a different regression model, in order to verify whether LEDD influences sleep quality, in correlation with pain, we found no significant association.

Multiple regression models were performed also to observe the contribution of other comorbidities (such as RLS, anxiety, and depression) on sleep and pain. RLS was found to have a significant impact on nocturnal pain and discoloration/edema/swelling (r^2^ = 0.35 and 0.115, respectively), among anxiety, depression, and sleep. Depression also influences musculoskeletal pain, among RLS, anxiety and sleep (r^2^ = 0.075).

## 4. Discussion

Longitudinal studies have demonstrated a bidirectional negative influence between sleep disturbances (especially insomnia) and pain [10]. Pain impacts quality of life in PD patients [24] and is also associated with other non-motor symptoms, such as restless legs syndrome [25], urinary dysfunction [26], and fatigue [27].

The pathophysiological mechanisms that may explain pain in Parkinson’s disease (PD) have not been entirely elucidated. Musculoskeletal and joint pain, frequently identified by PD patients, may be explained by the association with muscle rigidity, akinesia, and lack of mobility [28]. PD patients with pain have shown abnormal activity in PET scans during OFF stages in the structures responsible for the discriminatory perception and motivational/affective aspects of pain (e.g., in the insular somatosensory cortex or the anterior cingulate gyrus) [29]. Primary central pain in PD may have its substrate in dopaminergic dysfunction at the pain inhibition centers [29]. Neurodegenerative processes affecting dopaminergic and serotoninergic pathways in specific brainstem regions (such as raphe nuclei) may contribute to both abnormal pain perception and impaired sleep [30]. Pain often disrupts sleep in PD patients [31], and sleep duration may also modulate pain intensity. Although not specifically observed in patients with PD, studies have suggested that sleep deprivation may increase perceived pain in healthy individuals and possibly in patients with chronic pain [32]. Sleep deprivation (sleep duration of less than 6 h) can lead to hyperalgic complaints [33], and a low pain threshold may persist after REM sleep deprivation, despite the restoration of normal sleep [34].

The main findings of our study are as follows: 1. the most reported subtype of pain in our group was the musculoskeletal one; 2. the subtype of pain exhibited by patients can differ between genders; 3. as the severity of pain increases, the severity of motor status and the spectrum of sleep disorders also increase; 4. there is a bidirectional relationship between pain and sleep disorders, as “bad sleepers” report more pain compared to “good sleepers”; 5. the odds of PD patients presenting sleep disturbances are higher among patients with nocturnal pain, orofacial pain, and radicular pain. Although the insights of previous research regarding pain subtypes and other motor and non-motor symptoms are diverse, some of our findings are in accordance with the existent literature, aspects that will be further discussed in this section. Our results may differ from the previous ones due to the heterogeneity of the population included in the study and the different methods of assessment that we used.

Based on the domain scores of the KPPQ, the prevalence of various types of pain was assessed in this study. Musculoskeletal pain was the most frequently reported (82.44%), followed by nocturnal pain (58.77%) and radicular pain (55.72%). The results are comparable to those identified in the literature. According to the study conducted by Fu et al., the frequency order of pain types was as follows: musculoskeletal pain (80%), dystonic pain (16%), neuropathic pain (14.7%), central pain (10.7%), and other types of pain (9.3%) [8]. According to another study, 64.9% of enrolled patients reported pain, with the most common types being musculoskeletal pain (44.4%), dystonic pain (19.1%), central (12.7%), and radicular or neuropathic (11.1%) [35]. In another study, among the 176 PD patients investigated, 70% reported musculoskeletal pain [36]. Additionally, the pain subtype most frequently associated with sleep disturbances was musculoskeletal pain [11]. Ozturk et al. reported the following types of pain in a cohort of 113 PD patients: musculoskeletal pain (89.0%), radicular/peripheral pain (31.5%), dystonic pain (15.1%), and parkinsonian central pain (4.1%) [37]. Furthermore, a recent study demonstrated that musculoskeletal and nocturnal pain are the best predictors of sleep disturbances in PD, with an explanation involving possibly related pathophysiological mechanisms underlying dysfunctions at the level of the locus coeruleus (for pain) and the reticular formation and raphe nucleus (for sleep disturbances) [12].

According to the classification based on the severity of pain (as assessed with total mean scores of KPPS), most patients enrolled in this study reported mild pain (56.48%); no patient reported severe pain (KPPS score > 68). Unexplained pain is a commonly reported symptom by PD patients. A reference study regarding the prevalence of non-motor symptoms in PD patients reported a prevalence of pain in 29% of PD patients [38]. The DoPaMiP (Douleur et maladie de Parkinson en Midi-Pyrénées) study conducted in the southern regions of France found that 62% of PD patients complained of at least one type of chronic pain [39]. Other reported prevalences of pain were 69.6% [40], or 83% [36]. More than half of PD patients reported at least one type of pain, 24% reported two different types of pain, and 5% reported three distinct types of pain [36]. According to another study involving 113 patients, 35.6% of them reported experiencing different types of pain simultaneously [37]. The variations in the reported prevalences of pain can be explained by different inclusion criteria, the heterogeneity of the evaluated population, and the definition proposed for pain [35].

In the present study, patients with a higher degree of pain severity also exhibited more pronounced motor symptoms, according to the SCOPA—motor part and CISI assessments. Similar to the results of this study, PD patients with pain were older and had a longer duration of PD, a more advanced severity of PD, and higher LEDD compared to PD patients without pain [8]. There are also studies that have reported pain to be more prevalent in younger age groups [39]. Additionally, some studies have shown no association between pain and age, disease duration, or severity [29,36,41].

In our study, females reported more musculoskeletal pain, visceral pain, and pain related to burning mouth syndrome in comparison with males. Burning mouth syndrome was also observed with a higher prevalence in females compared to males in a previous study that included 198 PD patients [42]. In their study, Gao et al. reported that female gender is associated with chronic, pain related to motor fluctuations, orofacial pain and discoloration/edema/swelling [41].

Associations between pain severity and poor sleep quality were noted in this study, according to the total PSQI scores. Similar to our results, pain severity was also correlated with poor sleep quality, anxiety, depression, and longer disease duration in another cohort study that included 52 PD patients [43]. A multicenter study surveyed 300 PD patients, of which 99.3% reported at least one sleep disorder, according to the PDSS-2 assessment. A moderate-to-high correlation was observed between sleep, pain (evaluated by PDSS-2, KPPS, KPPQ and VAS-Pain), and quality of life (evaluated by EQ5D-3L and Parkinson’s Disease questionnaire-8—PDQ-8) [12].

In the current research, patients with sleep disturbances (“bad sleepers”, according to a PSQI score > 5) reported more severe pain compared to those without sleep disturbances (“good sleepers”), particularly in the KPPS domains related to musculoskeletal pain, chronic pain (i.e., central pain), nocturnal pain (i.e., pain related to difficulty turning in bed), and radicular pain. The odds of presenting sleep disturbances (based on a PDSS-2 score ≥ 18) are higher among patients with nocturnal pain, orofacial pain, and radicular pain, a finding also confirmed after adjusting for motor status (according to MDS-UPDRS motor part evaluation). According to the regression models performed in this study, different domains of pain (musculoskeletal, nocturnal and discoloration/edema/swelling) were correlated (even if in a modest manner) with RLS, anxiety, depression and sleep. Similar results were found in the study conducted by Gao et al., in which pain was analyzed using the KPPS scale [11]. Patients with poor sleep quality (PSQI < 5) had higher KPPS scores compared to patients without sleep disturbances for the following domains: musculoskeletal pain, chronic pain, fluctuation-related pain, nocturnal pain, and discoloration/edema/swelling. Furthermore, PSQI scores significantly correlated with the total KPPS score and with the seven component domains of the KPPS scale [11]. A study involving 45 PD patients analyzed the characteristics of nocturnal pain and its relationship with sleep, evaluated using the PDSS scale. According to the results, the average duration of uninterrupted sleep during the night was 3–4 h. Paresthesia and numbness were significantly associated with the highest VAS scores, suggesting that pain may be a significant cause of sleep fragmentation. Three female participants reported disturbing nighttime pain in this study. Other common causes for sleep fragmentation were nocturia and restless legs syndrome, but other types of pain were also noted, such as painful lower limb cramps, lumbago, limb/facial dystonia, or akinesia (difficulty mobilizing in bed) [31]. Another study conducted by Hilten et al. has shown that difficulties in maintaining continuous sleep were more common in PD patients, the most common causes for sleep fragmentation being nocturia, pain, stiffness, and akinesia [44]. According to one study, anxiety and depression represented the most relevant predictors of pain, which was evaluated using the visual analogue scale [12].

We acknowledge that our study has several limitations. This research lacks a control group, which could provide more precise insights into the specific characteristics of pain in PD patients compared to healthy individuals. A longitudinal design would offer a better understanding regarding the temporal relationship between pain occurrence and sleep disorders. Pain and sleep were assessed only using subjective scales and questionnaires; the influence of medication on pain and sleep was not assessed. Further research is required to confirm our results. Larger samples, longitudinal studies, polysomnographic analysis, and assessment of medication effects on pain and sleep may provide a better understanding of the bidirectional connection between pain and sleep, and may contribute to the development of targeted therapeutic interventions.

## 5. Conclusions

The main conclusions of our studies emphasize the fact that pain and sleep disturbances are common in PD patients and may share tight bidirectional causality. Moreover, pain severity correlates with the severity of sleep problems. As PD patients complaining of nocturnal, orofacial, and radicular pain have higher odds of sleep disturbances, it is important to screen and adequately manage pain in these patients.

## Figures and Tables

**Table 1:** Main demographic data of PD patients included in the study.

Age at PD onset, mean ± SD	68.30 ± 10.69
PD duration, years, mean ± SD	5.06 ± 4.21
LEDD, mg (mean ± SD)	497.2 ± 385.2
Dyskinesia, n (%)	7 (5.3%)
Dystonia, n (%)	6 (4.6%)
HY 1 ON, n (%)	7 (5.3%)
HY 2 ON, n (%)	78 (59.5%)
HY 3 ON, n (%)	34 (26%)
HY 4 ON, n (%)	11 (8.4%)
HY 5 ON, n (%)	1 (0.8%)

HY, Hoehn and Yahr stage; LEDD, levodopa equivalent daily dose; SD, standard deviation; PD, Parkinson’s disease.

**Table 2:** Main characteristics of “good sleepers” (PSQI score ≤ 5) compared to “bad sleepers” (PSQI > 5), in relation to the components of the King’s Parkinson’s Disease Pain Scale. Values include standard deviation.

	“Good Sleepers” (PSQI ≤ 5), n = 33 (25.19%)	“Bad Sleepers” (PSQI > 5), n = 98 (74.80%)	*p*
Age, years (mean ± SD)	66.52 ± 11.65	70.23 ± 9.40	0.067
Disease duration, years (mean ± SD)	4.47 ± 4.63	5.27 ± 4.07	0.351
Domain 1: musculoskeletal pain	2.70 ± 2.67	5.48 ± 3.50	**<0.001**
Domain 2: chronic pain	0.45 ± 1.12	1.73 ± 2.51	**0.005**
Central pain	0.15 ± 0.71	1.19 ± 2.01	**0.004**
Visceral pain	0.30 ± 0.59	0.54 ± 0.99	0.193
Domain 3: fluctuation-related pain	0.09 ± 0.52	0.38 ± 2.02	0.422
Dyskinetic pain	0.06 ± 0.35	0.07 ± 0.50	0.909
“off” period dystonia	0.03 ± 0.17	0.20 ± 1.17	0.397
“off” period pain	0.00 ± 0.00	0.10 ± 0.67	0.381
Domain 4: nocturnal pain	0.82 ± 1.59	2.78 ± 3.31	**0.001**
Restless legs syndrome	0.18 ± 0.53	0.52 ± 1.24	0.130
Pain related to difficulty turning in bed	0.64 ± 1.22	2.26 ± 2.74	**0.001**
Domain 5: orofacial pain	0.24 ± 1.09	0.57 ± 1.61	0.278
Pain when chewing	0.06 ± 0.35	0.20 ± 0.77	0.305
Grinding teeth	0.06 ± 0.35	0.08 ± 0.40	0.787
Burning mouth syndrome	0.12 ± 0.48	0.29 ± 0.91	0.323
Domain 6: discoloration/edema/swelling	2.33 ± 3.48	3.47 ± 4.08	0.154
Burning pain in limbs	2.24 ± 3.28	3.41 ± 3.95	0.129
Generalized lower abominal pain	0.09 ± 0.29	0.06 ± 0.32	0.635
Domain 7: radicular pain	2.45 ± 3.55	4.35 ± 4.20	**0.022**
KPPQ total score (mean ± SD)	3.09 ± 2.40	5.11 ± 3.41	**0.002**
KPPS total score (mean ± SD)	9.09 ± 10.25	18.76 ± 14.40	**0.001**

KPPQ, King’s Parkinson’s Disease Pain Questionnaire; KPPS, King’s Parkinson’s Disease Pain Scale; PSQI, Pittsburgh Sleep Quality Index. Bold values denote statistical significance (*p* < 0.05).

**Table 3:** Odds of PD patients with pain having sleep disturbances, in accordance with pain subtypes.

Pain Subtype (According to KPPQ)	Odds (95% CI)	*p*	Adjusted Odds *	*p*
Musculoskeletal pain	1.040 (0.958–1.128)	0.351	-	-
Chronic pain	1.052 (0.998–1.110)	0.061	-	-
Fluctuation-related pain	1.045 (0.991–1.102)	0.107	-	-
Nocturnal pain	1.161 (1.062–1.268)	**0.001**	1.165 (1.064–1.276)	**0.001**
Orofacial pain	1.069 (1.009–1.133)	**0.023**	1.108 (1.051–1.167)	**<0.001**
Discoloration/edema/swelling	1.059 (1.004–1.116)	**0.034**	1.05 (0.994–1.108)	0.079
Radicular pain	1.088 (1.024–1.156)	**0.007**	1.015 (1.015–1.149)	**0.015**

King’s Parkinson’s Disease Pain Questionnaire (KPPQ). Bold values denote statistical significance (*p* < 0.05). * The values are adjusted for severity of motor symptoms (MDS-Unified Parkinson’s Disease Rating Scale—UPDRS III).

## Data Availability

The data presented in this study are available on reasonable request from the corresponding author due to the data is not in a public repository.

## Abbreviations

The following abbreviations are used in this manuscript:

CISI	Clinical Impression of Severity Index
H&Y	Hoehn and Yahr
HADS	Hospital Anxiety and Depression Scale
KPPQ	King’s Parkinson’s Disease Pain Questionnaire
KPPS	King’s Parkinson’s Disease Pain Scale
LEDD	Levodopa equivalent daily dose
MDS-UPDRS	MDS-Unified Parkinson’s Disease Rating Scale
MMSE	The Mini Mental State Examination
MoCA	Montreal Cognitive Assessment
PD	Parkinson’s Disease
PDSS-2	Parkinson’s Disease Sleep Scale-2
PSQI	Pittsburgh Sleep Quality Index
RLS	Restless legs syndrome
SCOPA	Scale for Outcomes in Parkinson’s Disease
